# Blood lipid profiles, fatty acid deposition and expression of hepatic lipid and lipoprotein metabolism genes in laying hens fed palm oils, palm kernel oil, and soybean oil

**DOI:** 10.3389/fvets.2023.1192841

**Published:** 2023-07-13

**Authors:** Wan Ibrahim Izuddin, Teck Chwen Loh, Nazri Nayan, Henny Akit, Ahmadilfitri Md Noor, Hooi Ling Foo

**Affiliations:** ^1^Department of Animal Science, Faculty of Agriculture, Universiti Putra Malaysia, Serdang, Selangor, Malaysia; ^2^Institute of Tropical Agriculture and Food Security (ITAFoS), Universiti Putra Malaysia, Serdang, Selangor, Malaysia; ^3^Institute of Bioscience, Universiti Putra Malaysia, Serdang, Selangor, Malaysia; ^4^Sime Darby Plantation Research Sdn Bhd, R&D Centre – Carey Island, Carey Island, Selangor, Malaysia; ^5^Department of Bioprocess Technology, Faculty of Biotechnology and Biomolecular Science, Universiti Putra Malaysia, Serdang, Selangor, Malaysia

**Keywords:** crude palm oil, red palm oil, refined palm oil, palm kernel oil, soybean oil, medium-chain fatty acids, poultry, chicken

## Abstract

The palm oil, palm kernel oil and soybean oil have unique and distinctive fatty acid chain length and saturation profiles, and how they affect lipid peroxidation, fatty acid intake and metabolism is worth exploring in poultry. This study elucidated the influence the dietary oils on lipid peroxidation, blood lipid profiles, fatty acid deposition of liver, serum and yolk and the expression of liver genes related to lipid and lipoprotein metabolism in laying hens. About 150 Hisex brown laying hens were fed diets containing crude palm oil (CPO), red palm oil (RPO), refined palm oil (RBD), palm kernel oil (PKO) or soybean oil (SBO) for 16 weeks. Serum, liver and yolk lipid peroxidation were not different between dietary oils. The PKO increased liver, serum and yolk medium-chain fatty acids (MCFA). There was no difference in liver saturated fatty acids (SFA). The CPO and RPO reduced serum SFA, but the PKO increased yolk SFA. The SBO increased polyunsaturated fatty acids (PUFA) in liver serum and yolk. No difference in liver elaidic acid (C18:1-trans), but SBO lowered elaidic acid (C18:1-trans) in serum. Higher very-low density lipoprotein (VLDL) in CPO than RPO and SBO and greater serum lipase in CPO, RBD and PKO than SBO. There was no difference in sterol regulatory element-binding protein 2 (SREBP-II) between oils. Apolipoprotein VLDL-II (APOVLDL2) was upregulated in palm oils and apolipoprotein B-100 (APOB) in RBD. Downregulation in peroxisome proliferator-activated receptor-alpha (PPAR-α), peroxisome proliferator-activated receptor gamma (PPAR-γ) and low-density lipoprotein receptor (LDLR) was observed in palm oils and PKO. In conclusion, different dietary oils greatly influence several aspects of fatty acid metabolism, deposition and lipoprotein profiles but have no influence on reducing lipid peroxidation.

## 1. Introduction

Oils or fats are included in the diet of commercial laying hens at 2–3% to achieve 5% crude fat, as recommended by most commercial breeds. High oil inclusion contributed to excessive fat deposition, which reduced reproductive performance in laying hens (1, 2). Although oil is added at a small percentage in poultry feed, it significantly impacts poultry performance as it supplies a better composition of fatty acids in the diet (3). In addition, it improves the physical quality of feed by reducing dustiness, enhancing feed palatability, and supplying essential fat-soluble vitamins (4–6). Lipids can be sourced either from plant or animal based. Plant-based oil has advantages over animal-based oil due to the lower price with higher polyunsaturated fatty acids (PUFA), particularly omega-3 fatty acid and contains naturally occurring phytonutrients such as polyphenols, squalene, carotenoids and vitamins (7, 8).

The inclusion of oils supplies metabolically essential fatty acids such as omega-3 and omega-6 fatty acids to the poultry. The fatty acid profiles of the feed contributed by the oils are the critical factor in influencing the profile of fatty acid in the blood and fatty acid deposition in the body tissues and poultry products such as meat and eggs (3, 4, 9–14). Eggs enriched with omega-3 fatty acids through hens’ diets produce value-added eggs for human consumption that deliver anti-inflammatory properties to reduce health risks (15, 16). The composition of fatty acids characterized the oil, and fatty acid composition is the primary predictor of oxidative stability (17). The higher the amount of unsaturated fatty acids (USFA) fraction in oils, the higher and faster the oxidation process occurs compared to saturated fatty acids (SFA) (18). Lower USFA in oil is expected to reduce lipid peroxidation. Since different properties of oil sources have other effects on lipid metabolism, deposition and oxidation, selecting a source of oil to be added to the poultry feed is important.

**Figure 1:**
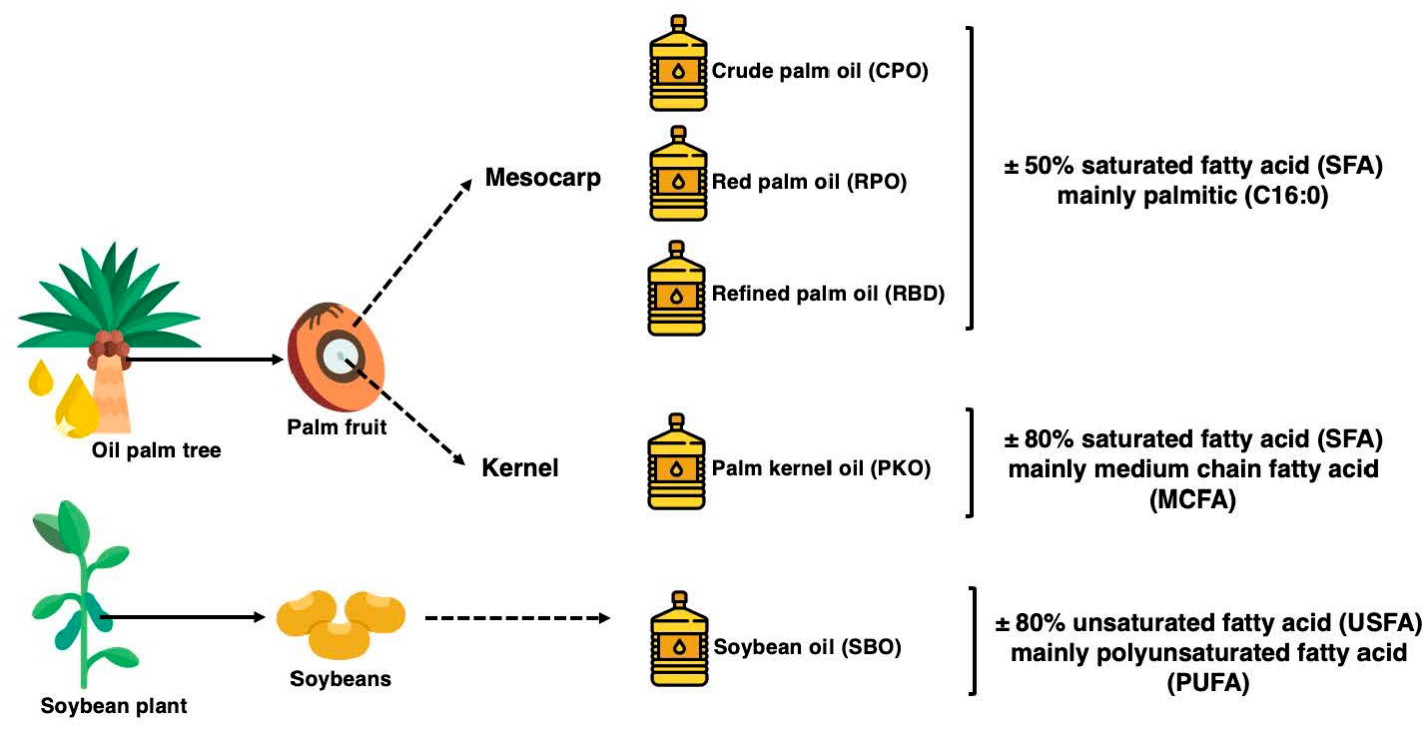
The summary of the source of the palm oils, palm kernel oil, and soybean oil and its saturation profiles adapted from (19–21).

Palm oils such as crude palm oil (CPO), red palm oil (RPO) and refined palm oil (RBD) are produced from the mesocarp of the palm fruit. It contains palmitic (C16:0) and oleic (C18:1)-rich fatty acids and is balanced in the SFA to USFA fraction. It also contains high levels of antioxidants contributed by vitamin E in RBD and both vitamin E and carotenoids in CPO and RPO. On the other hand, palm kernel oil (PKO) is extracted from the kernel of the palm fruit. PKO is rich in medium-chain fatty acids (MCFA), mainly lauric acid (C12:0) and myristic acid (C14:0) and contains up to 80% SFA. Soybean oil (SBO) is a polyunsaturated fatty acid (PUFA)-rich oil extracted from soybeans and undergoes refining, bleaching and deodorization to produce refined SBO. The summary of the source of oils and the fatty acid characteristics are displayed in Figure 1, which is adapted from (19–21).

Recently, the effects of dietary CPO, RPO, RBD, and PKO on production performance, egg quality, serum biochemicals and profiles of beta-carotene, retinol and tocopherols in laying hens were reported (22). Oils produced from oil palms, such as CPO, RPO, RBD, and PKO, were not previously studied with respect to fatty acid metabolism and serum lipid and lipoprotein profiles in laying hens. The unique properties of the fatty acid composition, saturation profiles, and naturally occurring antioxidant compounds such as vitamin E and carotenoids are worth exploring. Shorter and saturated fatty acids had higher antioxidant activity and reduced the degree of oxidation. Sengupta et al. (23) reported rice bran oil containing caprylic (C8:0), caproic (C10:0) or lauric (C12:0) increased antioxidant activity in all antioxidant assays. However, they had a lower thiobarbituric acid reactive substance (TBARS) value and conjugated diene as compared with the control rice bran oil, which contained a higher fraction of a longer chain of SFA mainly palmitic acid (C16:0) and USFA mainly oleic (C18:1) and linoleic (C18:2) acids. Hence, this study focused on comparing the dietary palmitic-rich CPO, RPO and RBD, MCFA-rich PKO and PUFA-rich SBO on the serum lipid profiles, the fatty acid composition of feed and its deposition in the serum, liver and yolks, and the liver lipid metabolism gene expression. In addition, the SBO was included as a reference oil with high PUFA as a comparison. It is hypothesized that the fatty acid profiles of oil will influence the blood lipid profiles, lipid peroxidation and deposition of fatty acid profiles in feeds, serum, liver and yolk in laying hens.

## 2. Materials and methods

### 2.1. Ethic approval, dietary treatments, and hens’ management

**Figure 2:**
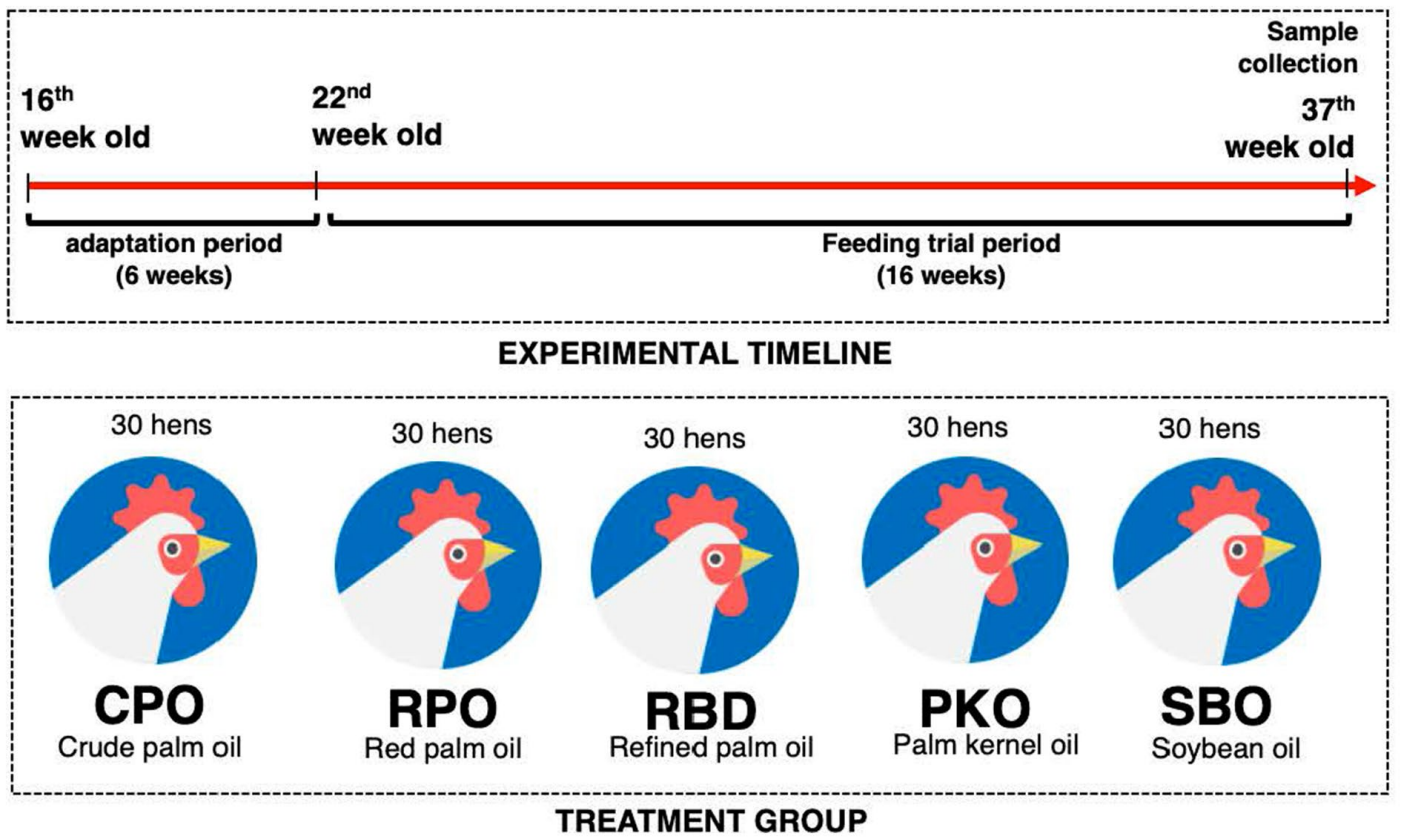
The summary of treatment groups and experimental timeline.

The experimental protocol was approved by the Institutional Animal Care and Use Committee of Universiti Putra Malaysia (AUP No: UPM/IACUC/AUP-R013/2020). The experimental location was at the Poultry Unit, Department of Animal Science, Universiti Putra Malaysia. A total of 150 Hisex Brown laying hens were randomized into five treatment groups (30 hens per group), which contained six biological replicates per treatment and five hens per biological replicate. The dietary treatments contained either 3% of CPO, RPO, RBD, PKO, or SBO. The diets were formulated to be isocaloric and isonitrogenous (Table 1) and to achieve the nutrient requirements of Hisex Brown laying hens. The feeds were prepared monthly. Each hen received 120 g feed daily in mash form, as recommended by the Hisex Brown nutritional guide. The water was given *ad libitum* through a nipple drinker. The feeding trial was from week 22 to week 37 (16 weeks). The housing system was an open-sided house, and temperature and humidity were 24 to 32°C and 80 ± 5%, respectively. Hens were placed in two-tier A-type battery cages (30 cm width, 50 cm depth and 40 cm height) individually and received a total of 16 h (±12 h of natural light and 4 h of LED fluorescent light) and 8 h of darkness (Figure 2).

**Table 1:** Ingredients and nutrient profiles of feeds containing different oils.

	CPO	RPO	RBD	PKO	SBO
Ingredients (%)					
Corn	48.90	48.90	48.90	48.90	48.90
Soybean meal	28.00	28.00	28.00	28.00	28.00
Wheat pollard	8.000	8.000	8.000	8.000	8.000
CPO	3.000	-	-	-	-
RPO	-	3.000	-	-	-
RBD	-	-	3.000	-	-
PKO	-	-	-	3.000	-
SBO	-	-	-	-	3.000
DL-Methionine	0.300	0.300	0.300	0.300	0.300
MDCP	2.300	2.300	2.300	2.300	2.300
Calcium carbonate	8.350	8.350	8.350	8.350	8.350
Choline chloride	0.200	0.200	0.200	0.200	0.200
Salt	0.350	0.350	0.350	0.350	0.350
Mineral mix	0.200	0.200	0.200	0.200	0.200
Vitamin mix	0.200	0.200	0.200	0.200	0.200
Antioxidants	0.100	0.100	0.100	0.100	0.100
Toxin binder	0.100	0.100	0.100	0.100	0.100
TOTAL	100.0	100.0	100.0	100.0	100.0
Calculated nutrient (in % unless stated)					
ME (kcal/kg)	2,790	2,790	2,790	2,790	2,790
CP	17.17	17.17	17.17	17.17	17.17
EE	4.980	4.98	4.98	4.98	4.98
*CF*	3.800	3.80	3.80	3.80	3.80
Ca	4.000	4.00	4.00	4.00	4.00
Total phosphorus	0.840	0.84	0.84	0.84	0.84
Avail. phosphorus	0.460	0.46	0.46	0.46	0.46
Methionine	0.581	0.581	0.581	0.581	0.581
Lysine	0.933	0.933	0.933	0.933	0.933

CPO, crude palm oil; RPO, red palm oil; RBD, refined palm oil; PKO, palm kernel oil; SBO, soybean oil; MDCP, mono dicalcium phosphate; ME, metabolizable energy; CP, crude protein; EE, ether extract; CF, crude fiber; Ca, calcium.

### 2.2. Sample collection and analysis

**Figure 3:**
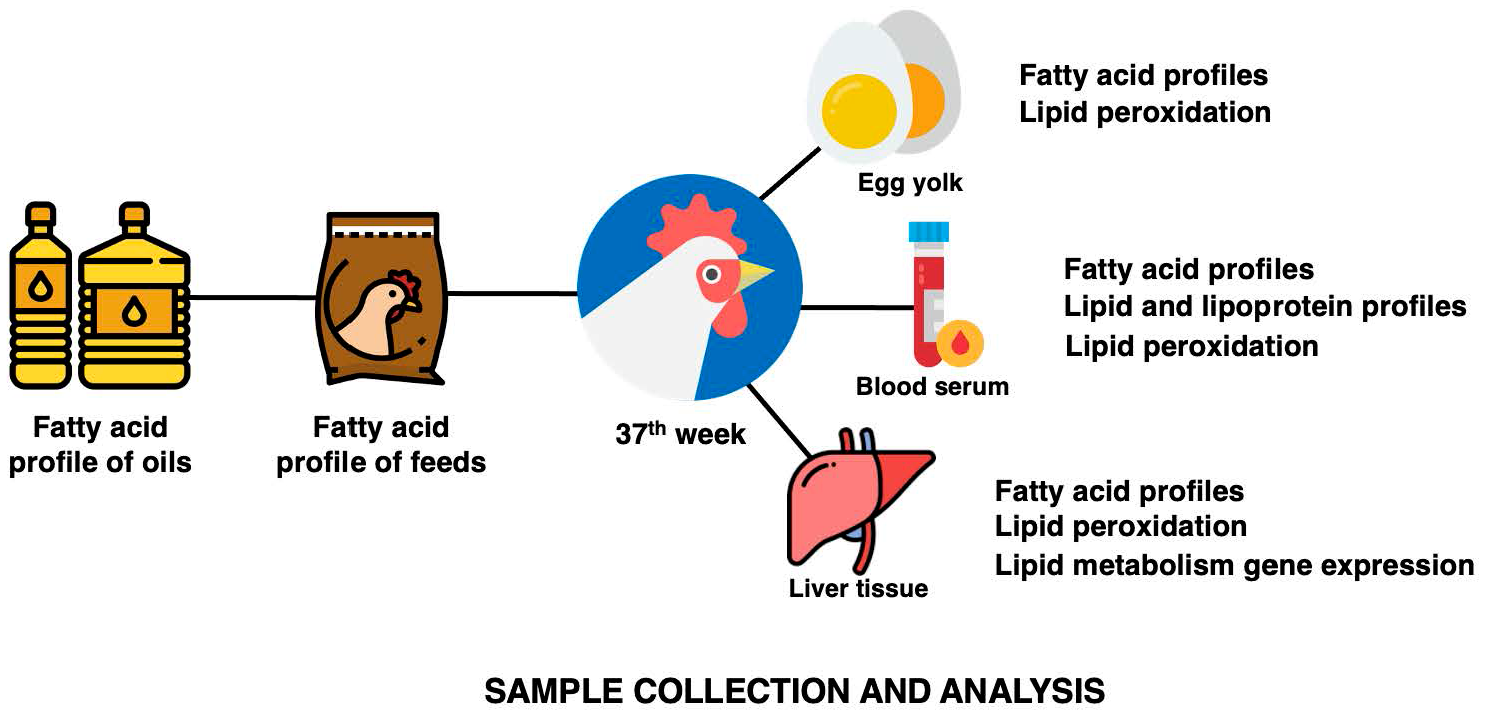
The summary of sample collection from oils, feeds, and hens and its analysis.

Feed samples were collected and kept in a −80°C freezer until analysis. Two eggs from each biological replicate were collected randomly at the end of the experimental period for egg yolk collection. Two yolks were homogenized, combined, freeze-dried at −84°C in a freeze dryer (Labconco, Kansas City, MO, USA), and kept at −80°C until analysis. At the end of the experimental period, six birds per treatment were randomly selected for sacrifice through Halal slaughter and sample collection. At the bleeding point, about 8 ml of blood was collected into a 10 ml BD Vacutainer® Serum Tubes tube (BD, Franklin Lakes, NJ, USA) and allowed to clot on ice. The serum was separated from the blood through centrifugation (3,000 RCF for 20 min at 4°C) and stored at −80°C. A portion of lower right lobe of the liver sample were collected at evisceration into a cryotube and snap-frozen before being kept at −80°C (Figure 3).

### 2.3. Serum lipid and lipoprotein profiles

The serum biochemistry was analyzed at the Veterinary Hematology and Clinical Biochemistry Laboratory, Faculty of Veterinary Medicine, Universiti Putra Malaysia. Serum samples were analyzed for cholesterol (TC), triacylglycerol (TAG), high-density lipoprotein (HDL), low-density lipoprotein (LDL) and lipase using the Roche/Hitachi 902 clinical chemistry automatic analyzer (Roche, Basel, Switzerland) using appropriate kits. The very-low-density lipoprotein (VLDL) in serum was determined using a Chicken VLDL ELISA kit (FineTest, Wuhan, Hubei, China) according to the protocol provided by the manufacturer. The ELISA kit was based on a double antibody to capture and detect the target protein. The absorbance was determined at 450 nm using an ELx800™ microplate reader (BioTek™, Winooski, Vermont, USA) equipped with Gen5 Microplate Reader and Imager Software (BioTek™, Winooski, VT, USA). The concentration of VLDL was interpolated based on the constructed standard curve of different concentrations of VLDL (μg/ml) against absorbance at 450 nm.

### 2.4. Lipid peroxidation

Lipid peroxidation was determined using a thiobarbituric acid reactive substance (TBARS) assay by measuring malondialdehyde (MDA) as the product of lipid peroxidation in serum, liver and yolk samples. One gram of sample or 1 ml of MDA standard was combined with 4 ml of 1.15% (w/v) potassium chloride and mixed by the vortex. The mixture was added to 2.5 ml TBARS solution comprising 2 ml of 0.8% (w/v) thiobarbituric acid in 20% (v/v) acetic acid at pH 3.5, 300 μl of deionized water, 165 μl of 8.1% (w/v) SDS and 35 μl 7.0 ethanolic butylated hydroxytoluene. The mixture was mixed by vortex, incubated in a water bath at 95°C for 60 min, and cooled down to room temperature. The cooled mixture was added to 3 ml of n-butanol, mixed by vortex and centrifuged at 5000 rpm for 10 min at 24°C. The supernatant was collected into a cuvette to read absorbance at 532 nm using a SPECORD® 250 PLUS UV/Vis spectrophotometer (Analytic Jena, Jena, Germany). The lipid peroxidation was calculated from the linear regression derived from a standard curve of MDA and expressed as the concentration of MDA per gram sample.

### 2.5. Fatty acid profile of oil, feed, liver, serum, and egg yolk

Fifty milligrams of the oil sample were combined with 950 μl hexane and 50 μl of 1 N methanolic sodium methoxide and mixed by a vortex. The mixture was allowed to stand for 5 min and centrifuged at 3000 × *g* for 5 min for complete separation. The upper layer was collected into a 2.0 ml glass vial with a PTFE screw cap for fatty acid methyl ester (FAME) separation. For feed, liver, egg yolk (1 g) and serum (1 ml), the sample was mixed with 10 ml of 2:1 (v/v) chloroform: methanol containing butylated hydroxytoluene in a glass tube with a PTFE-lined screw cap, mixed by inversion and stood for 12 h in the dark at 4°C. Five milliliters of 0.9% (w/v) sodium chloride were added, mixed by vortex and centrifuged at 3000 × *g* for 5 min at 24°C. The lower phase was collected into a fresh glass tube with a PTFE-lined screw cap containing 100 μl of 12 mM methanolic heneicosanoic acid (C21:0) (Sigma-Aldrich, St. Louis, MI, USA) before incubating in a 70°C water bath under a constant and mild flow of nitrogen gas to evaporate chloroform. The tubes were cooled to room temperature, and 2 ml of 0.66 N methanolic potassium hydroxide was added into the tubes, mixed by vortex, screwed tightly and incubated in 90°C water for 10 min. The tubes were cooled to room temperature, and 2 ml of 20% methanolic boron trifluoride was added into the tubes, mixed by vortex, screwed tightly and incubated in a 90°C water bath for 20 min. The tubes were cooled to room temperature, and 4 ml of deionized water and 4 ml of petroleum ether were added. The tubes were mixed by vortex and centrifuged at 3000 × *g* for 5 min at 24°C. About 1 ml of upper phase (petroleum ether) was aliquoted into a 2.0 ml glass vial with a PTFE-lined screw cap to be injected into gas chromatography.

Fatty acids were determined using an Agilent 6,890 N Network Gas Chromatograph (Agilent, Santa Clara, CA, USA) equipped with an autosampler and injector. About 1 μl of the sample was injected into the inlet set at 250°C with a split mode of 30:1. The flame ionization detector was set at 250°C with a hydrogen gas flow of 40 ml/min, air flow of 450 ml/min and make up the flow of 45 ml/min. The nitrogen gas was used as a carrier gas with a constant flow rate of 1 ml/min. The oven temperature gradient was set at 80°C and held for 2 min, increased at 5°C/min to 150°C and held for 10 min, increased at 4°C/min to 220°C and held for 10 min at 220°C with a total runtime of 53 min. The fused-silica capillary column used was a J&W HP-88 GC Column (112-8867), 60 m, 0.25 mm, and 0.20 μm in film thickness (Agilent, CA, USA). The heneicosanoic acid (C21:0; Sigma-Aldrich, St. Louis, MI, USA) was used as an internal standard, and the 37 Component FAME Mix (Merck, Darmstadt, Germany) was used as an external standard to identify the target fatty acids. The SFA is the sum of caprylic (C8:0), caproic (C10:0), lauric (C12:0), myristic (C14:0), palmitic (C16:0) and stearic (C18:0) acids. The USFA is the sum of palmitoleic (C16:1), oleic (C18:1), linoleic (C18:2), and linolenic (C18:3) acids. The MUFA is the sum of palmitoleic (C16:1) and oleic (C18:1) acids. The PUFA is the sum of linoleic (C18:2) and linolenic (C18:3) acids.

### 2.6. Liver’s lipid metabolism genes

Total RNA was extracted using a NucleoSpin® RNA plus kit (Machery Nagel, Dueren, Germany). The quantity and quality of extracted RNA were confirmed using the spectrophotometry method on a Multiskan™ Go spectrophotometer (Thermo Scientific, Waltham, MA, USA). The reverse transcription (1 μg RNA) was conducted using the cDNA Synthesis Kit (Biotechrabbit, Berlin, Germany). The qPCR reaction comprised 1 μl cDNA, 1 μl each for forward and reversed primers, 5 μl 4× CAPITAL™ qPCR Green Master Mix (Biotechrabbit, Berlin, Germany) and 12 μl nuclease-free water. The reaction was conducted using the LightCycler® 480 Instrument (Roche, Basel, Switzerland). The cycling program setting was initially activated at 95°C for 2 min and 30 s, followed by 45 cycles of quantification steps containing a denaturation step at 95°C for 15 s. Then, it was followed by combined annealing and extension for 30 s at a temperature specific to the primer. The specificity of the amplification was confirmed using a melt curve. The information for housekeeping and target genes is available in Table 2. The expression of the target gene was calculated using Livak’s 2^−∆∆Ct^ method (24).

**Table 2:** The forward and reverse of primer sequence, product size, accession number of target genes.

Target gene	Primer sequence	Product size (bp)	Accession number
GAPDH F	CTGGCAAAGTCCAAGTGGTG	275	NM_204305.1
GAPDH R	AGCACCACCCTTCAGATGAG		
APOB F	AGGTGGTGGTGAAGAGGTGGAGAG	97	NM_001044633.1
APOB R	GAGCAGCAAGAGCCGCACAG		
PPAR-α F	TGCTGTGGAGATCGTCCTGGTC	166	NM_001001464.1
PPAR-α R	CTGTGACAAGTTGCCGGAGGTC		
PPAR-γ F	TACATAAAGTCCTTCCCTCTGACC	470	NM_001001460.1
PPAR-γ R	TCCAGTGCATTGAACTTCACAGC		
SREBP-2 F	CCCAGAACAGCAAGCAAGG	108	XM_040660556.1
SREBP-2 R	GCGAGGACAGGAAAGAGAGTG		
apoVLDL2 F	ATGGTGCAATACAGGGCATT	196	NM_205483.2
apoVLDL2 R	GGGAAACATCCAGCAAGAAC		
LDLR F	CGCGTCCGGCTCCATATC	457	NM_204452.1
LDLR R	CTCGCAGCCCCACTCATCC		

GAPDH, glyceraldehyde 3-phosphate dehydrogenase; APOB, apolipoprotein B-100; PPAR-α, peroxisome proliferator-activated receptor-alpha; PPAR-γ, peroxisome proliferator-activated receptor gamma; SREBP2, sterol regulatory element-binding protein 2; APOVLDL2, apolipoprotein VLDL-II; LDLR, low-density lipoprotein receptor; F, forward; R, reverse.

### 2.7. Experimental design and statistical analysis

The feeding trial was subjected to a completely randomized design (CRD). The data analysis was performed on the SAS software package, version 9.4 (SAS Inst. Inc., Cary, NC, USA). The data normality was determined using PROC UNIVARIATE and determined based on Shapiro–wilk. All data obtained were normally distributed and analyzed using one-way analysis of variance (ANOVA) using the General Linear Model (GLM) procedure and paired with Duncan’s multiple-range test for comparing the treatment means. The difference was considered significant at *p* < 0.05.

## 3. Results

### 3.1. Fatty acid profiles of oils

Different types of oils showed significant differences (*p* < 0.05) in their fatty acid profiles (Table 3). The PKO contained the significantly highest (*p* < 0.05) fraction of medium-chain fatty acids (MCFA), which include caprylic (C8:0), caproic (C10:0), lauric (C12:0) and myristic (C14:0) acids compared to other treatments. The significantly highest (*p* < 0.05) SFA was present in PKO (81.86%), followed by CPO (60.87%), RPO (57.04%), RBD (53.58%) and SBO (28.54%). However, USFA and PUFA fractions were contrary to the SFA, in which SBO had the significantly highest (*p* < 0.05) and PKO the lowest (*p* < 0.05). MUFA had the significantly highest (*p* < 0.05) percentage in RBD (40.79%), followed by RPO (37.73%), CPO (34.39%), SBO (29.88%) and the lowest (*p* < 0.05) in PKO (16.72%). The ratio of USFA to SFA, MUFA to SFA and PUFA to SFA was significantly highest (*p* < 0.05) in SBO, followed by RBD, RPO, CPO and PKO.

**Table 3:** The fatty acid profiles of oils.

	CPO	RPO	RBD	PKO	SBO	SEM	*p*-value
Caprylic acid (C8:0)	0.019^b^	0.014^b^	0.010^b^	2.680^a^	0.009^b^	0.285	<0.001
Caproic acid (C10:0)	0.021^b^	0.019^b^	0.015^b^	2.776^a^	0.006^b^	0.296	<0.001
Lauric acid (C12:0)	0.521^b^	0.452^b^	0.392^b^	44.61^a^	0.422^b^	4.722	<0.001
Myristic acid (C14:0)	0.900^b^	0.856^b^	0.843^b^	16.20^a^	0.152^c^	1.660	<0.001
Palmitic acid (C16:0)	55.54^a^	51.85^b^	49.80^c^	13.44^e^	22.37^d^	4.606	<0.001
Stearic acid (C18:0)	3.833^b^	3.812^b^	3.547^c^	2.373^d^	5.529^a^	0.270	<0.001
Oleic acid (C18:1-cis)	34.37^c^	37.71^b^	40.76^a^	16.71^e^	29.84^d^	2.249	<0.001
Linoleic acid (C18:2n-6)	4.606^d^	5.087^c^	5.564^b^	1.420^e^	37.22^a^	3.554	<0.001
Linolenic acid (C18:3n-3)	0.126^c^	0.139^b^	0.069^d^	0.003^e^	4.302^a^	0.451	<0.001
SFA	60.87^b^	57.04^c^	53.58^d^	81.86^a^	28.54^e^	4.557	<0.001
USFA	39.13^d^	42.96^c^	46.42^b^	18.14^e^	71.46^a^	4.557	<0.001
MUFA	34.39^c^	37.73^b^	40.79^a^	16.72^e^	29.88^d^	2.251	<0.001
PUFA	4.735^d^	5.230^c^	5.637^b^	1.424^e^	41.58^a^	4.010	<0.001
USFA:SFA	0.643^d^	0.753^c^	0.867^b^	0.222^e^	2.504^a^	0.210	<0.001
MUFA:SFA	0.565^d^	0.662^c^	0.762^b^	0.204^e^	1.047^a^	0.073	<0.001
PUFA:SFA	0.078^d^	0.092^c^	0.105^b^	0.017^e^	1.457^a^	0.148	<0.001

CPO, crude palm oil; RPO, red palm oil; RBD, refined palm oil; PKO, palm kernel oil; SBO, soybean oil; SFA, saturated fatty acid; USFA, unsaturated fatty acid; MUFA, monounsaturated fatty acid; PUFA, polyunsaturated fatty acid. ^a,b,c,d,e^Means with different superscripts in the same row depict significant differences (*p* < 0.05). Experimental unit, *n* = 3.

### 3.2. Fatty acid profiles of feeds

There were significant differences (*p* < 0.05) in the fatty acid profiles of the feed containing different oils (Table 4). The SFA of feed was significantly highest (*p* < 0.05) in PKO, followed by CPO, RPO and RBD, and the lowest (*p* < 0.05) fraction was in SBO. The USFA had an inverse trend to the SFA in which SBO had the significantly highest (*p* < 0.05) fraction, followed by RBD and RPO, CPO and the lowest (*p* < 0.05) in PKO. The significantly highest (p < 0.05) fraction of MUFA was found in RPO and RBD, with no difference (*p* > 0.05) between each other, followed by CPO, SBO and PKO. Total PUFA and linoleic acid (C18:2n-6) concentrations were significantly higher (P0.05) in SBO and lower (P0.05) in PKO. In palm oils (CPO, RPO and RBD), there is a significantly higher (*p* < 0.05) total PUFA and linoleic acid (C18:2n-6) in RPO compared to CPO, with no difference (*p* > 0.05) between RPO and RBD or between CPO and RBD. The linolenic acid (C18:3n-3) had the significantly highest (*p* < 0.05) concentration in SBO compared to other oils. The ratio of USFA to SFA was significantly highest (*p* < 0.05) in SBO, followed by RBD and RPO, CPO and the lowest (*p* < 0.05) ratio in PKO.

**Table 4:** Fatty acid profiles of feeds containing different oils.

	CPO	RPO	RBD	PKO	SBO	SEM	*p*-value
Caprylic acid (C8:0)	0.012^b^	0.000^b^	0.000^b^	2.245^a^	0.036^b^	0.240	<0.001
Caproic acid (C10:0)	0.010^b^	0.000^b^	0.000^b^	1.976^a^	0.035^b^	0.210	<0.001
Lauric acid (C12:0)	0.264^b^	0.206^b^	0.171^b^	27.95^a^	0.476^b^	2.962	<0.001
Myristic acid (C14:0)	0.637^c^	0.600^c^	0.578^c^	9.527^a^	5.654^b^	0.973	<0.001
Palmitic acid (C16:0)	45.57^a^	41.80^b^	41.82^b^	18.26^d^	23.62^c^	2.962	<0.001
Stearic acid (C18:0)	3.948^b^	3.852^b^	3.851^b^	3.226^b^	4.875^a^	0.167	0.0086
Oleic acid (C18:1)	33.85^b^	35.85^a^	36.46^a^	21.85^d^	28.89^c^	1.467	<0.001
Linoleic acid (C18:2n-6)	14.91^c^	17.80^b^	16.27^bc^	14.18^c^	34.95^a^	2.090	<0.001
Linolenic acid (C18:3n-3)	0.798^b^	0.894^b^	0.850^b^	0.780^b^	3.234^a^	0.260	<0.001
SFA	50.44^b^	46.46^c^	46.42^c^	63.19^a^	34.10^d^	2.507	<0.001
USFA	49.56^c^	53.54^b^	53.58^b^	36.81^d^	65.90^a^	2.507	<0.001
MUFA	33.85^b^	35.85^a^	36.46^a^	21.85^d^	28.40^c^	1.480	<0.001
PUFA	15.71^cd^	18.20^b^	17.12^bc^	14.96^d^	37.50^a^	2.278	<0.001
USFA:SFA	0.983^c^	1.155^b^	1.154^b^	0.583^d^	1.934^a^	0.118	<0.001

CPO, crude palm oil; RPO, red palm oil; RBD, refined palm oil; PKO, palm kernel oil; SBO, soybean oil; SEM, standard error of means; SFA, saturated fatty acid; USFA, unsaturated fatty acid; MUFA, monounsaturated fatty acid; PUFA, polyunsaturated fatty acid; PUFA3, omega-3 fatty acids; PUFA6, omega-6 fatty acids. ^a,b,c,d^Means with different superscripts in the same rows depict significant differences (*p* < 0.05). Experimental unit, *n* = 3.

### 3.3. Lipid profiles and lipase enzyme activity of serum

There were no significant differences (*p* > 0.05) in the serum cholesterol (TC), triacylglycerol (TGL), low-density lipoprotein (LDL) and high-density lipoprotein (HDL; Table 5). There were significant differences (*p* < 0.05) in the concentration of very low-density lipoprotein (VLDL) and lipase enzyme activity. Significantly higher (*p* < 0.05) lipase enzyme activity was observed in the CPO, RBD, and PKO compared to the SBO. There was a significant difference (*p* < 0.05) in lipase enzyme activity between RPO and SBO. The serum VLDL was significantly affected (*p* < 0.05) by different dietary supplementations of oils. Significantly higher (*p* < 0.05) serum VLDL concentrations were observed in CPO as compared to RPO and SBO but not different (*p* > 0.05) to RBD and PKO. Serum VLDL did not significantly differ (*p* > 0.05) between RPO, RBD, PKO and SBO.

**Table 5:** Serum lipid profiles and lipase in laying hens fed different oils.

Treatment	CPO	RPO	RBD	PKO	SBO	SEM	*p*-value
TC (mmol/L)	3.463	3.079	2.521	3.184	2.883	0.158	0.4610
TAG (mmol/L)	8.741	8.714	8.622	8.389	8.731	0.076	0.6243
LDL (mmol/L)	0.974	0.915	0.803	0.977	0.871	0.049	0.8365
HDL (mmol/L)	0.999	0.859	0.846	1.093	0.925	0.055	0.6692
VLDL (μg/ml)	0.803^a^	0.579^b^	0.611^ab^	0.659^ab^	0.539^b^	0.033	0.0490
Lipase (U/L)	41.33^a^	38.66^ab^	41.92^a^	41.95^a^	34.68^b^	0.901	0.0131

CPO, crude palm oil; RPO, red palm oil; RBD, refined palm oil; PKO, palm kernel oil; SBO, soybean oil. SEM, standard error of means; TC, cholesterol; TAG, triacylglycerol; LDL, low-density lipoprotein; HDL, high-density lipoprotein. ^a,b^Means with different superscripts in the same rows depict significant differences (*p* < 0.05). Experimental unit, *n* = 6.

### 3.4. Lipid peroxidation in serum, yolk, and liver

The TBARS value of serum, yolk and liver did not differ between different oils (Table 6).

**Table 6:** Thiobarbituric acid reactive substance (TBARS) of serum, yolk, and liver in laying hens fed different oils.

Treatment	CPO	RPO	RBD	PKO	SBO	SEM	*p*-value
Serum (μg/ml MDA)	3.118	4.929	5.169	2.802	4.935	0.411	0.185
Yolk (μg/g MDA)	53.36	53.36	43.41	43.74	54.02	1.835	0.099
Liver (μg/g MDA)	42.03	40.85	32.14	45.95	36.28	1.768	0.089

CPO, crude palm oil; RPO, red palm oil; RBD, refined palm oil; PKO, palm kernel oil; SBO, soybean oil; SEM, standard error of means. Experimental unit, *n* = 6.

### 3.5. Fatty acid profiles of liver

There were significant differences (*p* < 0.05) in most of the profiles except for palmitic (C16:0) and elaidic (C18:1-trans) acids, total SFA, USFA, and the ratio of USFA to SFA (Table 7). Dietary supplementations of PKO significantly increased (*p* < 0.05) lauric (C12:0) and myristic (C14:0) acids. There was no significant difference (*p* > 0.05) in lauric acid (C12:0) between CPO, RPO and RBD or between SBO and RPO. There was no significant difference (*p* > 0.05) in myristic acid (C14:0) between CPO, RPO, RBD and SBO. Significantly higher (*p* < 0.05) stearic (C18:0), linoleic (C18:2n-6) and linolenic (C18:3n-3) acids were found in SBO than other oils. Significantly higher (*p* < 0.05) MUFA fraction in RPO with no difference (*p* > 0.05) in RBD and lowest (*p* < 0.05) in PKO and SBO. The SBO showed higher total PUFA, linolenic acid (C18:3n-3) and linoleic acid (C18:2n-6) than other dietary supplementations of oils.

**Table 7:** Fatty acid profiles of the liver in laying hens fed different oils.

	CPO	RPO	RBD	PKO	SBO	SEM	*p*-value
Lauric acid (C12:0)	0.138^c^	0.191^bc^	0.118^c^	0.691^a^	0.333^b^	0.045	<0.0001
Myristic acid (C14:0)	0.544^b^	0.558^b^	0.492^b^	2.215^a^	0.503^b^	0.124	<0.0001
Palmitic acid (C16:0)	41.15	38.83	41.13	40.76	38.31	0.414	0.0569
Palmitoleic acid (C16:1)	1.206^abc^	1.000^bc^	1.239^ab^	1.582^a^	0.753^c^	0.081	0.0101
Stearic acid (C18:0)	13.60^b^	12.39^b^	13.29^b^	13.61^b^	16.35^a^	0.381	0.0138
Elaidic acid (C18:1-trans)	0.613	0.476	0.765	0.669	0.779	0.070	0.6754
Oleic acid (C18:1)	33.37^bc^	37.61^a^	34.91^ab^	31.48^cd^	29.46^d^	0.708	0.0003
Linoleic acid (C18:2n-6)	5.872^b^	7.388^b^	6.311^b^	6.539^b^	11.814^a^	0.464	<0.0001
Linolenic acid (C18:3n-3)	0.129^b^	0.188^b^	0.142^b^	0.132^b^	0.484^a^	0.031	<0.0001
SFA	55.43	51.96	55.02	57.58	54.67	0.661	0.1054
USFA	44.57	48.04	44.98	42.88	45.33	0.661	0.1853
MUFA	35.35^b^	39.09^a^	36.91^ab^	33.73b^c^	30.99^c^	0.710	0.0005
PUFA	7.656^b^	8.948^b^	8.069^b^	8.685^b^	14.34^a^	0.511	<0.0001
USFA:SFA	0.812	0.925	0.821	0.737	0.846	0.022	0.1116

CPO, crude palm oil; RPO, red palm oil; RBD, refined palm oil; PKO, palm kernel oil; SBO, soybean oil; SEM, standard error of means; SFA, saturated fatty acid; USFA, unsaturated fatty acid; MUFA, monounsaturated fatty acid; PUFA, polyunsaturated fatty acid. PUFA3, omega-3 fatty acids; PUFA6, omega-6 fatty acids; TRANS, trans fatty acids. ^a,b,c,d,e^Means with different superscripts in the same rows depict significant differences (*p* < 0.05). Experimental unit, *n* = 6.

### 3.6. Fatty acid profiles of serum

All fatty acid profiles showed a significant difference (*p* < 0.05) between treatment groups (Table 8). In MCFA, lauric acid (C12:0) was significantly higher (*p* < 0.05) in SBO, and myristic acid (C14:0) was the highest (*p* < 0.05) in PKO. The stearic acid (C18:0) was significantly higher (*p* < 0.05) in RBD, PKO and SBO than in CPO and RPO. The SFA was significantly higher (*p* < 0.05) in RBD, PKO and SBO than in CPO and RPO. The USFA was significantly higher (*p* < 0.05) in CPO and RPO and lower (*p* < 0.05) in RBD, PKO and SBO. The MUFA was significantly higher (*p* < 0.05) in CPO and RPO, followed by RBD and PKO and lowest (*p* < 0.05) in SBO. The total PUFA, linolenic acid (C18:3n-3) and linoleic acid (C18:2n-6) were significantly higher (*p* < 0.05) in SBO compared to other oils. The trans fatty acid (elaidic acid; C18:1-trans) was significantly lower (*p* < 0.05) in SBO. Serum USFA:SFA was significantly higher (*p* < 0.05) in CPO and RPO than in RBD, PKO and SBO.

**Table 8:** Fatty acid profiles of serum in laying hens fed different oils.

	CPO	RPO	RBD	PKO	SBO	SEM	*p*-value
Lauric acid (C12:0)	0.094^c^	0.582^b^	0.133^bc^	1.862^a^	0.355^bc^	0.137	<0.0001
Myristic acid (C14:0)	0.481^c^	0.692^bc^	0.429^c^	2.035^a^	0.911^b^	0.123	<0.0001
Palmitic acid (C16:0)	40.78^bc^	39.75^c^	42.18^ab^	43.28^a^	41.57^abc^	0.361	0.0145
Palmitoleic acid (C16:1)	1.456^ab^	1.276^bc^	1.156^bc^	1.788^a^	0.869^c^	0.087	0.0061
Stearic acid (C18:0)	11.16^b^	11.15^b^	13.66^a^	13.30^a^	13.94^a^	0.365	0.0117
Elaidic acid (C18:1-trans)	1.050^a^	0.981^a^	0.913^a^	1.026^a^	0.740^b^	0.029	0.0008
Oleic acid (C18:1)	39.12^a^	39.27^a^	35.68^ab^	33.01^bc^	30.28^c^	0.813	<0.0001
Linoleic acid (C18:2n-6)	5.710^b^	6.146^b^	5.678^b^	6.017^b^	9.420^a^	0.278	<0.0001
Linolenic acid (C18:3n-3)	0.146^b^	0.154^b^	0.172^b^	0.156^b^	0.410^a^	0.020	<0.0001
SFA	52.52^b^	52.18^b^	56.40^a^	58.45^a^	58.28^a^	0.717	0.0013
USFA	47.48^a^	47.82^a^	43.60^b^	43.27^b^	41.72^b^	0.619	0.0004
MUFA	41.62^a^	41.52^a^	37.75^b^	35.44^b^	31.89^c^	0.849	<0.0001
PUFA	5.856^b^	6.300^b^	5.850^b^	6.117^b^	9.830^a^	0.297	<0.0001
USFA:SFA	0.904^a^	0.918^a^	0.775^b^	0.719^b^	0.723^b^	0.022	0.0004

CPO, crude palm oil; RPO, red palm oil; RBD, refined palm oil; PKO, palm kernel oil; SBO, soybean oil; SEM, standard error of means; SFA, saturated fatty acid; USFA, unsaturated fatty acid; MUFA, monounsaturated fatty acid; PUFA, polyunsaturated fatty acid; PUFA3, omega-3 fatty acids; PUFA6, omega-6 fatty acids. ^a,b,c^Means with different superscripts in the same rows depict significant differences (*p* < 0.05). Experimental unit, *n* = 6.

### 3.7. Fatty acid profiles of egg yolk

There were significant differences (*p* < 0.05) in the yolk fatty acid profiles between the treatments (Table 9). The lauric (C12:0) and myristic (C14:0) acids were significantly higher (*p* < 0.05) in PKO. The SFA was significantly higher (*p* < 0.05) in PKO, followed by CPO, SBO, RPO, and the lowest (*p* < 0.05) in RBD, with no difference (*p* > 0.05) between SBO with CPO and RPO. The USFA was significantly highest (*p* < 0.05) in RBD, followed by CPO, RPO and lowest (*p* < 0.05) PKO. The SBO had no difference (*p* > 0.05) from the CPO and RPO in the USFA. The MUFA was significantly highest (*p* < 0.05) in RBD, followed by CPO and RPO, PKO, and the lowest (*p* < 0.05) in SBO. The total PUFA, linolenic acid (C18:3n-3) and linoleic acid (C18:2n-6) were significantly higher (*p* < 0.05) in SBO than in other oils. The USFA:SFA was significantly higher (*p* < 0.05) in RBD and lowest (*p* < 0.05) in PKO. There was no significant difference (*p* > 0.05) in SBO compared to CPO and RPO. No trans fatty acids (elaidic acid; C18:1-trans) were detected in the egg yolk.

**Table 9:** Fatty acid profiles of egg yolk in laying hens fed different oils.

	CPO	RPO	RBD	PKO	SBO	SEM	*p*-value
Lauric acid (C12:0)	0.521^b^	0.562^b^	0.490^b^	0.833^a^	0.434^b^	0.047	0.0348
Myristic acid (C14:0)	0.493^b^	0.445^b^	0.415^b^	2.949^a^	0.399^b^	0.269	<0.001
Palmitic acid (C16:0)	40.62^a^	39.87^ab^	38.84^c^	40.16^a^	39.18^ac^	0.196	0.0029
Palmitoleic acid (C16:1)	2.085^b^	1.926^b^	1.862^b^	2.511^a^	1.546^c^	0.088	<0.001
Stearic acid (C18:0)	7.366^b^	7.182^b^	7.492^b^	7.591^b^	8.601^a^	0.150	0.0024
Oleic acid (C18:1)	42.41^b^	43.03^b^	44.09^a^	39.07^c^	36.54^d^	0.757	<0.001
Linoleic acid (C18:2n-6)	6.346^b^	7.102^b^	6.637^b^	6.679^b^	12.62^a^	0.644	<0.001
Linolenic acid (C18:3n-3)	0.158^c^	0.188^bc^	0.174^bc^	0.207^b^	0.678^a^	0.053	<0.001
SFA	49.00^b^	48.06^c^	47.24^d^	51.53^a^	48.61^bc^	0.397	<0.001
USFA	51.00^c^	51.94^b^	52.76^a^	48.47^d^	51.39^bc^	0.397	<0.001
MUFA	44.50^b^	44.96^b^	45.95^a^	41.58^c^	38.09^d^	0.771	<0.001
PUFA	6.504^b^	6.985^b^	6.811^b^	6.886^b^	13.30^a^	0.701	<0.001
USFA:SFA	1.041^c^	1.081^b^	1.117^a^	0.940^d^	1.057^bc^	0.016	<0.001

CPO, crude palm oil; RPO, red palm oil; RBD, refined palm oil; PKO, palm kernel oil; SBO, soybean oil; SEM, standard error of means; SFA, saturated fatty acid; USFA, unsaturated fatty acid; MUFA, monounsaturated fatty acid; PUFA, polyunsaturated fatty acid; PUFA3, omega-3 fatty acids; PUFA6, omega-6 fatty acids. ^a,b,c,d^Means with different superscripts in the same rows depict significant differences (*p* < 0.05). Experimental unit, *n* = 6.

### 3.8. Liver lipid metabolism genes

The significant difference (*p* < 0.05) in the regulation of gene expression between oils was observed in apolipoprotein B-100 (APOB), peroxisome proliferator-activated receptor-alpha (PPAR-α), peroxisome proliferator-activated receptor gamma (PPAR-γ), apolipoprotein VLDL-II (apoVLDL2) and low-density lipoprotein receptor (LDLR) genes (Table 10). However, the sterol regulatory element-binding protein 2 (SREBP-II) gene did not significantly differ (*p* > 0.05) in expression. The APOB was significantly upregulated (*p* < 0.05) in RBD and had no difference (*p* > 0.05) in regulation in other types of oils. Significantly lower (*p* < 0.05) expression of PPAR-α, PPAR-γ and LDLR was observed in the palm oil groups (CPO, RPO and RBD) relative to SBO. The apoVLDL2 was significantly upregulated (*p* < 0.05) in CPO, RPO and RBD compared to PKO and SBO. There was no significant difference (*p* > 0.05) in apoVLDL2 expression between PKO and SBO.

**Table 10:** Liver lipid metabolism gene expression in laying hens fed different oils in relative to SBO.

Treatment	CPO	RPO	RBD	PKO	SBO	SEM	*p*-value
APOB	1.228^bc^	1.386^b^	1.973^a^	0.266^c^	1.000^bc^	0.225	0.005
PPAR-α	0.257^b^	0.273^b^	0.395^b^	0.479^b^	1.000^a^	0.079	0.001
PPAR-γ	0.022^c^	0.120^c^	0.129^c^	0.311^b^	1.000^a^	0.096	<0.001
SREBP-II	1.143	1.199	1.304	0.819	1.000	0.121	0.805
apoVLDL2	7.780^a^	5.088^a^	6.325^a^	2.029^b^	1.000^b^	0.894	0.001
LDLR	0.052^c^	0.130^c^	0.222^bc^	0.380^b^	1.000^a^	0.095	<0.001

CPO, crude palm oil; RPO, red palm oil; RBD, refined palm oil; PKO, palm kernel oil; SBO, soybean oil; SEM, standard error of means; APOB, apolipoprotein B-100; PPAR-α, peroxisome proliferator-activated receptor-alpha; PPAR-γ, peroxisome proliferator-activated receptor gamma; SREBP2, sterol regulatory element-binding protein 2; APOVLDL2, apolipoprotein VLDL-II; LDLR, low-density lipoprotein receptor. ^a,b,c^Means with different superscripts in the same rows depict significant differences (*p* < 0.05). Experimental unit, *n* = 6.

## 4. Discussion

### 4.1. Fatty acid profiles of oils and feeds

There was a reduction in the percentage of palmitic (C16:0), stearic (C18:0), linolenic (C18:3) and SFA and an increase in oleic (C18:1-cis), linoleic (C18:2), USFA, MUFA and PUFA across the CPO, RPO and RBD. Removal of the solid fraction (palm stearin) from CPO to obtain the liquid fraction (palm olein) to produce RPO and RBD would be the primary cause of the fatty acid composition shift. Palm stearin fraction had a higher SFA and lower USFA fraction than palm olein (25). The further fractionation processes of CPO to RPO through molecular distillation and CPO to RBD through refining, bleaching and deodorization also impacted the fatty acid profiles of the oils. It is worth noting that PKO had the highest SFA, mainly in the form of MCFA, compared to palm oils (CPO, RPO and RBD). The PKO originated from the kernel and has a different fatty acid profile composition than oil extracted from the mesocarp of the palm fruit such as CPO, RPO and RBD. The composition of omega-3 (linolenic acid; C18:3n-3) and omega-6 (linoleic acid; C18:2n-6) fatty acids in the oils and their inclusion in the diet of poultry to promote performance, health, and healthier products are receiving great attention. The SBO had superior characteristics in terms of n-3 and n-6 fatty acid content compared to palm oils (CPO, RPO and RBD) and PKO. Therefore, the presence of higher omega-3 and omega-6 fatty acids in oils and the poultry diet is beneficial to the birds and may yield poultry products such as meat and eggs enriched with n-3 and n-6 fatty acids and concurrently deliver the benefits to consumers (26).

The fatty acid profile of feeds was greatly dependent on the oil source. Khatun et al. (9) reported that adding palm oil and SBO to the diet of poultry influences the fatty acid composition of the feeds. Feed containing palm oils (CPO, RPO and RBD) had higher SFA contributed by higher palmitic (C16:0) and stearic (C18:0) acids. The increment was contributed by the higher percentage of palmitic acid (C16:0) in about 50–55% of palm oil (19, 27). Khatun et al. (9) also reported an increase in SFA and palmitic acid (C16:0) in the feed containing palm oil compared to SBO. Feed with PKO inclusion had a higher 63% SFA contributed by palmitic acid (C16:0) and MCFA such as lauric acid (C12:0) and myristic acid (C14:0). It was contributed by PKO, which contains higher MCFA, mainly lauric acid (C12:0) and myristic acid (C14:0) (27). The addition of SBO in the feed enhanced the USFA and PUFA fractions up to 66 and 38%, respectively, mainly due to oleic acid (C18:1), linoleic acid (C18:2n-6) and linolenic acid (C18:3n-3). The increment was contributed by higher USFA, PUFA, oleic acid (C18:1) and linoleic acid (C18:2n-6) in the SBO (19). Khatun et al. (9) also reported an increase in USFA, PUFA, oleic acid (C18:1) and linoleic acid (C18:2n-6) in the feed containing SBO as compared to palm oils.

### 4.2. Lipid profile and lipase enzyme activity of serum

The dietary supplementation of different oils did not influence the lipid biomarkers such as TC, TAG, LDL and HDL but affected VLDL. Our results corroborated Agboola et al. (28), who reported no difference in serum TAG, HDL, LDL and TC between laying hens fed 1.5% palm oil and 1.5% SBO. Furthermore, Agboola et al. (29) reported no difference in egg yolk TC, TAG, LDL, HDL, and VLDL between laying hens fed 1.5% palm oil and 1.5% SBO. However, Yifei et al. (30) reported no difference in serum cholesterol between 3% RPO and 3% SBO but lower serum TAG in 3% RPO compared to 3% SBO in laying ducks. A previous study showed that the increment in cholesterol increased as the level of oil increased. Kolani et al. (31) offered RBD at different levels from 0, 1, 2, to 3% in laying hens and found no effects on serum TAG, but TC was higher at 3% compared to 0 and 1% inclusion levels. Thus, the lack of difference in serum CHOL concentration in the current study could be linked to the similar inclusion of oil in the diet. Dietary fatty acids contribute to cholesterol synthesis, and an increase in dietary fatty acids contributes to higher cholesterol synthesis (31).

The liver synthesizes the VLDL and carries triacylglycerols and cholesterol in the blood for supply to the body’s tissues. The serum VLDL concentrations in the current study were higher in CPO than in RPO and SBO. The reasons for the higher serum VLDL between CPO and SBO could be related to the higher SFA contributed by long-chain fatty acids such as palmitic (C16:0) and stearic (C18:0) in CPO compared to the lower SFA in SBO. Similarly, Khatun et al. (4) found higher serum VLDL concentrations in broiler chickens fed 6% palm oil compared to 6% SBO. Crespo and Esteve-Garcia (32) also found higher serum VLDL concentrations in broiler chickens supplemented with tallow rich in SFA (50%), mainly palmitic (C16:0) and stearic (C18:0) acids compared to high USFA and PUFA-rich oils such as olive, sunflower and linseed oils. Despite higher SFA in the diet of PKO, there was no difference in serum VLDL concentration compared to SBO. The SFA in PKO was comprised of MCFA such as caprylic acid (C8:0), capric acid (C10:0), lauric acid (C12:0) and myristic acid (C14:0) which may contribute to the lack of difference in serum VLDL in the SBO. Khatun et al. (4) suggested dietary PUFA reduced chylomicron secretion in intestinal cells and reduced the synthesis of fatty acids and triacylglycerols in the liver. Thus, an increase in the fatty acid saturation in the diet of chickens contributed to the increase in fatty acid synthesis and fat deposition (9).

In addition, the serum lipase was higher in palm oils (CPO and RBD) and PKO than in SBO. The contribution of the increment in serum lipase enzyme activity may be correlated to the SFA in palm oils and PKO. The SFA in palm oil is rich in C16:0 (palmitic acid) and the SFA in PKO is rich in MCFA in the form of lauric acid (C12:0) and myristic acid (C14:0). Lipase is an enzyme in the blood plasma that hydrolyses triacylglycerol into free fatty acids and glycerol. Dietary SFA increased the serum VLDL that carries triacylglycerols in the blood (9, 32) and increased serum lipase enzyme activity for the deposition of triacylglycerols in tissues.

### 4.3. Lipid peroxidation in serum, yolk, and liver

The secondary lipid peroxidation products, such as MDA, indicate lipid oxidation and oxidative stress (33). A higher concentration of lipid oxidation products indicates higher oxidation, leading to an increase in inflammation and oxidative stress (34). Naturally occurring antioxidant compounds, such as vitamins, react with oxidants to prevent further oxidation and reduce oxidative stress. Tavárez et al. (35) The addition of in-feed antioxidants prevented further oxidizing of lipids, improved broiler performance and enhanced the meat’s shelf life even with dietary oxidized oil. Abdulla et al. (36) reported a higher concentration of MDA in the breast meat of broiler chickens fed high USFA oils such as SBO and linseed oil than palm oil.

However, the current study revealed that different oils did not affect the lipid peroxidation in serum, yolk and liver despite the differences in fatty acid composition and the presence of antioxidants such as vitamin E and carotenoids in the feed. It could be linked to the similar concentration of antioxidants such as retinol and tocopherol in the serum, liver and yolk of laying hens fed CPO, RPO, RBD, PKO and SBO (22). The lack of effects may also be attributed to a similar inclusion level of oil and the sufficient protection capacity provided by the antioxidants and vitamins in the feed. However, the increase in the lipid content in the diet increased the lipid oxidation of the yolk, as Yeasmin et al. (37) reported that the increase in dietary CPO from 1.5, 3 to 5% markedly increased the lipid peroxidation of the egg yolk.

### 4.4. Fatty acid profiles of liver

Higher SFA mainly from palmitic acid (C16:0) in palm oil and higher MCFA from lauric acid (C12:0) and myristic acid (C14:0) in PKO did not contribute to the difference in liver SFA and palmitic acid (C16:0). Conversely, higher USFA in the diet of SBO did not contribute to higher liver USFA. The lack of difference in the palmitic acid (C16:0) in the liver was similar to that in the serum. However, Khatun et al. (38) found higher liver SFA and palmitic acid (C16:0) in broiler chickens fed palm oil compared to SBO. The fatty acids supplied by dietary oil influence the fatty acid deposition in the liver tissue of broiler chickens (9, 38). Our finding on the lack of effects despite the difference in fatty acid saturation of different oils was attributed to *de novo* fatty acid production in the liver, which determines the regulation of fatty acid production. The predominant fatty acids in the liver are palmitic (C16:0) and stearic (C18:0) acids for SFA, oleic (C18:1) and palmitoleic (C16:1) acids for MUFA and linoleic (C18:2n-6) and linolenic (C18:3n-3) acids for PUFA (39).

Dietary supplementation of PKO increased the MCFA in the liver, such as lauric acid (C12:0) and myristic acid (C14:0). The increment of MCFA in the liver was similar to the higher MCFA in the serum. No previous study reported the effects of dietary PKO on liver fatty acid profiles in laying hens. The increase could be related to higher lauric acid (C12:0) and myristic acid (C14:0) in PKO (27), which contributed to the higher deposition of such fatty acids in the liver.

There was no difference in USFA in the liver between different oils, but PUFA, linoleic acid (C18:2n-6) and linolenic acid (C18:3n-3) were higher in SBO. The increment of liver PUFA, linoleic acid (C18:2n-6) and linolenic acid (C18:3n-3) in the liver was similar to that in the serum. Similarly, Khatun et al. (38) found higher liver PUFA, linoleic acid (C18:2n-6) and linolenic acid (C18:3n-3) in broiler chickens fed SBO compared to palm oil. Despite SBO contributing high USFA to the diet, the lack of difference in liver USFA was contributed by the higher values of liver palmitoleic acid (C16:1) and oleic acid (C18:1) in palm oils and PKO and the higher values of liver stearic acid (C18:0) in SBO that balanced the USFA. The increase in liver PUFA, linoleic acid (C18:2n-6) and linolenic acid (C18:3n-3) in the SBO group was contributed by the higher percentages of linoleic acid (C18:2n-6) and linolenic acid (C18:3n-3) in SBO (9).

### 4.5. Fatty acid profiles of serum

Fatty acids present in the serum are composed of digested and absorbed fatty acids from the intestinal tract and *de novo* synthesis of fatty acids by the liver. Dietary supplementation of palm oils (CPO, RPO and RBD) did not affect serum palmitic acid (C16:0) compared to SBO. However, serum SFA and stearic acid (C18:0) were higher in PKO and SBO than in palm oil (CPO and RPO). Higher feed SFA and stearic acid (C18:0) in PKO contributed to the increase in serum SFA and stearic acid (C18:0). However, no difference in serum palmitic acid (C16:0) between oils and higher serum stearic acid in SBO was contributed by *de novo* synthesis of fatty acids in the liver. The liver synthesizes palmitic (C16:0) and stearic (C18:0) acids for SFA, oleic (C18:1) and palmitoleic (C16:1) acids for MUFA, and linoleic (C18:2n-6) and linolenic (C18:3n-3) acids for PUFA as the primary fatty acids for the body (39).

The PKO contributed to an increment in serum lauric (C12:0) and myristic (C14:0) acids. The increment of MCFA in the serum was similar to the higher MCFA in the liver. No previous study reported the effects of dietary PKO on serum fatty acid profiles in laying hens. The increase could be related to higher levels of lauric acid (C12:0) and myristic acid (C14:0) in PKO (27) that were absorbed into the blood and contributed to the higher levels of these fatty acids in the serum. Dietary supplementation of SBO contributed to lower elaidic acid (C18:1-trans) and higher PUFA, linolenic acid (C18:3n-3) and linoleic acid (C18:2n-6) than palm oils (CPO, RPO and RBD) and PKO. The increase in fatty acids in the serum was consistent with the higher levels of fatty acids in the diet and in the liver. The SBO contained higher USFA, PUFA, oleic acid (C18:1) and linoleic acid (C18:2n-6) (19) and contributed them to the diet. Khatun et al. (9) also reported an increase in USFA, PUFA, oleic acid (C18:1) and linoleic acid (C18:2n-6) in the feed containing SBO as compared to palm oils.

### 4.6. Fatty acid profiles of yolk

There were no differences in yolk palmitic acid (C16:0) between palm oil (CPO, RPO and RBD) and PKO to SBO despite the higher palmitic acid (C16:0) in the feed containing palm oils. Khatun et al. (9) reported higher levels of palmitic acid (C16:0) in feed containing palm oil than SBO. The lack of difference in yolk palmitic acid (C16:0) was consistent with the lack of difference in serum and liver palmitic acid (C16:0). Similar to serum and liver; there was no difference in yolk palmitic acid (C16:0) between oils and the higher yolk palmitic acid in SBO was contributed by the *de novo* synthesis of fatty acids in the liver that regulates the production of each fatty acid. The liver is synthesizing palmitic (C16:0) and stearic (C18:0) acids for SFA, oleic (C18:1) and palmitoleic (C16:1) acids for MUFA and linoleic (C18:2n-6) and linolenic (C18:3n-3) acids for PUFA as the main fatty acids for the body (39).

Dietary-specific fatty acids and *de novo* regulation of fatty acid production and packaging of lipoprotein in the liver determined the composition of fatty acid profiles in the egg yolk. Dietary supplementation with PKO increased the deposition of the lauric (C12:0) and myristic (C14:0) acids in egg yolk. The increment in the deposition of lauric (C12:0) and myristic (C14:0) acids in the yolk was consistent with the high lauric (C12:0) and myristic (C14:0) acids in the feed, liver and serum of PKO. The PKO is known to contain high levels of lauric acid (C12:0) and myristic acid (C14:0) (27).

Similarly, dietary supplementation of SBO contributed to the increment of egg yolk stearic acid (C18:0), PUFA, linolenic acid (C18:3n-3) and linoleic acid (C18:2n-6). The increase was consistent with the higher PUFA, PUFA, linolenic acid (C18:3n-3) and linoleic acid (C18:2n-6) in the feed, serum and liver containing SBO. The SBO contained higher levels of stearic acid (C18:0), PUFA, oleic acid (C18:1) and linoleic acid (C18:2n-6) (19), and the inclusion of SBO increased USFA, PUFA, oleic acid (C18:1) and linoleic acid (C18:2n-6) in the feed (9). However, Agboola et al. (29) reported no difference in yolk palmitic (C16:0), stearic (C18:0), oleic (C18:1), linoleic (C18:2n-6) and linolenic (C18:3n-3) acids, total SFA, MUFA and PUFA in laying hens fed 1.5% palm oil (RBD) and 1.5% SBO. The lower inclusion level of RBD and SBO might have less effect on the yolk fatty acid profiles due to the liver’s *de novo* regulation of fatty acid production (39).

### 4.7. Liver lipid metabolism genes

The PPAR-α regulates lipid metabolism in the liver, including fatty acid oxidation to produce energy. The presence of fatty acids activates the PPAR-α, which triggers β-oxidation for ketogenesis enhancement and adenosine triphosphate production (40). The PPAR-γ is highly expressed in adipocytes and lower in the liver and muscle. High expression of PPAR-γ in the liver may induce the emergence of lipid droplets due to the regulation of several proteins related to the uptake and storage of triacylglycerols (40, 41). In this study, liver PPAR-α and PPAR-γ showed a similar trend of downregulation in palm oils and PKO relative to the SBO. Higher regulation of PPAR-α and PPAR-γ in SBO might be attributed to the higher PUFA fraction in the diet contributed by SBO. Ramiah et al. (42) observed upregulation of liver PPAR-α, PPAR-γ, and liver fatty acid-binding protein (L-FABP) genes in broiler chickens supplemented with conjugated linoleic acids. The results suggested the prominent role of PPARs as a vital regulator in the chicken’s liver lipid metabolism.

Apolipoprotein B-100 is a major lipoprotein in chicken VLDL that permits the attachment of VLDL, intermediate lipoprotein (IDL) and LDL in the bloodstream to specific receptors on the cell surface (43, 44). The attachment allows the lipoprotein content to be endocytosed into the cell. The current study found that the liver APOB gene was highly regulated in RBD and lowest regulated in PKO, suggesting the contribution of long-chain SFA in inducing higher surface APOB protein production for VLDL, IDL and LDL. This finding was concurrent with higher expression of apoVLDL-2 in palm oils, which co-exists with APOB on VLDL but at a higher number. ApoVLDL-2 is the major apoprotein in VLDL. In laying hens, VLDL is primarily transported from the liver to supply triacylglycerols and cholesterol to the oocytes for subsequent use in the development of the embryo, and apoVLDL2 protein is present in a larger amount than APOB on the VLDL surface (45). Our results revealed that palm oils contributed to higher expression of the liver apoVLDL2 gene and similar gene regulation between PKO and SBO. Therefore, palmitic acid-rich diets of CPO, RPO and RBD contributed to the higher production of apoVLDL2 mRNA and would be linked to the higher production of VLDL. Previous studies showed that broiler chicken fed dietary palm oil (4) and tallow (32) increased serum VLDL compared to a diet with high PUFA.

The SREBP-II regulates the synthesis and cellular uptake of fatty acids and cholesterol. The SREBPB activates the LDLR for cholesterol uptake, and SREBP activates the acetyl-CoA carboxylase and fatty acid synthase for fatty acid synthesis (46). We found similar regulation in the SREBP-II gene between different dietary oils, indicating no effect of fatty acid composition on the SREBP-II gene regulation. However, the current study showed a difference in LDLR expression between different dietary oils. LDLR presents on the cell surface and recognizes ApoB 100 and Apo E and is an essential mediator of the cell to endocytose LDL, chylomicron remnants and IDL that determine the blood plasma concentration of LDL (47). Despite similar regulation of the SREBP-II gene, the LDLR gene was downregulated in palm oils and PKO, with the lowest regulation in CPO and RPO. Therefore, higher regulation of the LDLR gene in SBO might be associated with greater uptake of LDL by the liver to regulate the cholesterol concentration in blood plasma and cholesterol metabolism (48).

## 5. Conclusion

This study contributed to the knowledge of the influence of feeding palm oil, palm kernel oil and soybean oil with different fatty acid compositions on fatty acid metabolism, and blood lipid profiles in laying hens. It can be concluded that the inclusion of oil greatly influenced the fatty acid composition of the feed, and dietary supplementation with different oils had similar trends in determining fatty acid profiles in the serum, liver and egg yolk. All sources of oils are suitable to be used in laying hens’ diet, as no negative effects were observed in the fatty acid profiles, blood lipid profile and lipid peroxidation. The SBO has the advantage of increasing omega-3 and omega-6 fatty acids in the body’s tissues. Palm oils did not affect the SFA profiles, but PKO increased the SFA profiles contributed by MCFA. The selection of oils in the diet should be influenced by the target of the producer, such as increasing specific fatty acids in eggs and meats or reducing the cost of feed by choosing cheaper oils such as CPO.

## Data availability statement

The original contributions presented in the study are included in the article/supplementary files. Further inquiries can be directed to the corresponding author.

## Ethics statement

The animal study was reviewed and approved by Institutional Animal Care and Use Committee of Universiti Putra Malaysia (AUP No: UPM/IACUC/AUP-R013/2020).

## Author contributions

WI, TL, HA, NN, AN, and HF designed the experiment. WI carried out the experiment, laboratory work, data analysis and manuscript writing. TL, HA, NN, AN, and HF were involved with manuscript revision. All authors contributed to the article and approved the submitted version.

## Conflict of interest

The authors declare that the research was conducted in the absence of any commercial or financial relationships that could be construed as a potential conflict of interest.

## Publisher’s note

All claims expressed in this article are solely those of the authors and do not necessarily represent those of their affiliated organizations, or those of the publisher, the editors and the reviewers. Any product that may be evaluated in this article, or claim that may be made by its manufacturer, is not guaranteed or endorsed by the publisher.
